# Developing a Self-Administered Questionnaire as a Guide to Consultations with Women Treated for Breast Cancer

**DOI:** 10.1155/2011/390692

**Published:** 2011-05-23

**Authors:** Moyez Jiwa, Wendy Chan She Ping-Delfos, Kathy Briffa, Jill Sherriff, Gareth Merriman, Janice Nockolds, Liz Jardine, Toni Musiello, Glenys Longman

**Affiliations:** ^1^Curtin Health Innovation Research Institute, Faculty of Health Sciences, Curtin University, GPO Box U1987, Perth,WA 6845, Australia; ^2^School of Physiotherapy, Curtin University, GPO Box U1987, Perth, WA 6845, Australia; ^3^School of Public Health, Curtin University, GPO Box U1987, Perth, WA 6845, Australia; ^4^Ability Plus Therapy, 233, Berrigan Drive, Jandakot, WA 6164, Australia; ^5^School of Surgery M507 QEII Medical Centre, The University of Western Australia, 35, Stirling Highway, Crawley, WA 6009, Australia; ^6^(Royal Perth Hospital), Level 2, MRF Building, GPO Box X 2213, Perth, WA 6847, Australia

## Abstract

*Background*. Health professionals, including general practitioners involved in followup of breast cancer patients, need to systematically assess opportunities to offer patients support with ongoing or new problems. *Methods*. A self-administered needs assessment questionnaire was developed with reference to a multidisciplinary team. Short, evidence-based, readable questions were emphasized, and questions were tested for face validity. The questions flowed across three domains: physical, social, and psychological. Content validity and user friendliness were assessed. *Results*. A final set of 30 questions was rated as easy to read and comprehend (Flesch Reading Ease score 65.8 and Flesch-Kincaid Grade Level 6.9). When piloted with twenty-one patients the self-administered questionnaire detected 121 items of unmet need encompassing all three domains. *Conclusions*. This self-administered questionnaire has the potential to assist in the holistic assessment of breast cancer patient after treatment. The clinical value of the self-administered questionnaire will need to be further tested before it can be widely adopted.

## 1. Introduction

Most women who are diagnosed with breast cancer will survive five or more years after diagnosis, and in many countries breast cancer patients attend follow-up clinics for several years following treatment [1]. The value of follow-up care is based on proactive screening for conditions that are amenable to intervention in order to maximize the prospects for survival and high quality of life. Most investigations carried out or treatment offered to patients is guided by the symptoms or problems reported to practitioners. A recent study reported that most patients did not volunteer their symptoms or problems following breast cancer to their general practitioner, despite consulting that practitioner, but did report significant problems to their breast care nurse when systematically questioned [2]. This paper is a first step in developing a tool which may be of value in clinical practice. Health professionals may benefit from a self-administered questionnaire to assist with consulting patients about whether further treatment is indicated [3]. To our knowledge such a self-administered questionnaire has not been published. 

As an overall focus for the self-administered questionnaire the team adopted theories which purport that a breast cancer diagnosis is an “extraordinary” life event which brings about a shift in identity for women [4]. Therefore, we incorporated theories on embodiment from medical sociology by including questions on changes in physical appearance and functioning, relationships, interests, and overall well-being [5]. This incorporates the concept of biographical disruption [6–8], biographical reinforcement [9], and biographical reinvention [10]. These theories predict that the woman is likely to experience a shift in perspective following the diagnosis and treatment, and therefore, one might anticipate that some people with breast cancer struggle to adjust after what may have been a traumatic life event [11]. Patients often do not volunteer problems unless prompted, perhaps because they are unaware that effective treatment or support is available or they consider that long-term dysfunction is an inevitable outcome of breast cancer treatment. We aim to devise a self-administered questionnaire to help proactively screen patients for physical, psychological, and social dysfunction following treatment for breast cancer.

## 2. Methodology

A team of relevant health care professionals was assembled including members of the following specialties, numbers in brackets refer to number of representatives:

- general practice (1),
- dietetics (2),
- nursing (1),
- occupational therapy (2),
- physiotherapy (1),
- psychology (1),
- sexology (2).

The self-administered questionnaire builds on a pre-existing interview self-administered questionnaire developed locally. Each member of the team proposed a series of questions that might detect ongoing or new problems for the patient. The team was asked to identify the evidence as the basis for the questions. There was an emphasis on short, readable questions. The team was assembled at a workshop to review the proposed questionnaire. The questions were examined for face validity and tested for readability. The survey and rationale for the questions were shared in the team in round robin fashion until there was consensus on the final version of the self-administered questionnaire. Implementation theory suggests that factors to be taken into account in the planning of a new tool should include its potential compatibility with existing routines [12]. In other words the tool should be practical and readily assimilated into the routines of clinical practice. The tool was therefore piloted with 21 breast cancer patients attending a hospital clinic 1–4 years after treatment to test for content validity. The pilot testing was also aimed at testing the user friendliness and to assess if the self-administered questionnaire helped to screen for possible problems for each patient in any of the domains. Responses to the self-administered questionnaire were reviewed by the team and recommendations made for interventions based on individual responses. The predictive validity of the self-administered questionnaire will be further tested in a follow-up study in which patients will be offered interventions with reference to the self-administered questionnaire. 

## 3. Results

**Figure 1:**
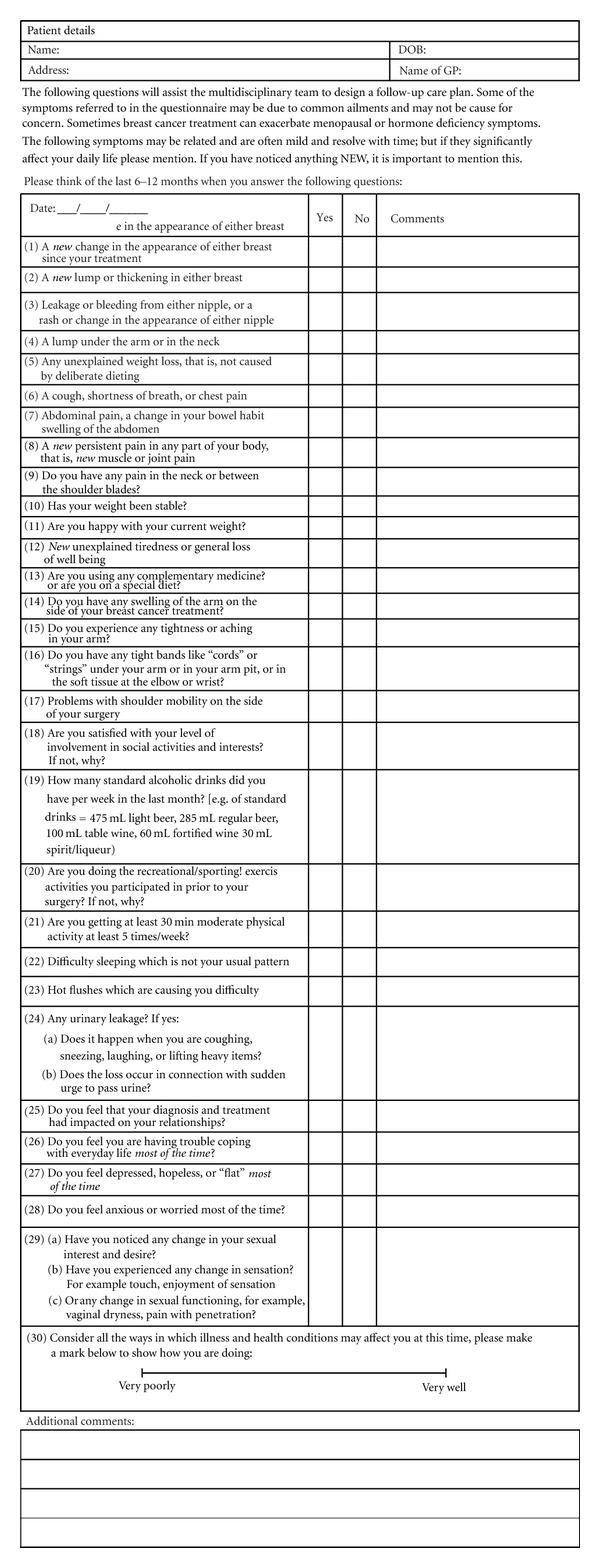
CCC (Cancer Care Coordinator) project—follow-up assessment.

For each dimension the following specific clinical evidence was incorporated into the questions by each member of the multidisciplinary team. The sequence of questions was reassigned to ensure a logical flow across the domains of interest (see Figure 1). The Flesch Reading Ease score [31] was 65.8, and the Flesh-Kincaid Grade Level was 6.9 [32]. The specific justification for the questions per specialty is recorded in Table 1. A key function in followup is to rule out recurrence of cancer and to assess for side effects of adjuvant therapy including tiredness, musculoskeletal complaints, weight gain, sexual dysfunction, gynaecologic symptoms, vasomotor symptoms, and urinary incontinence [33]. Urinary incontinence is a condition that can be treated effectively [22, 23], and yet care-seeking is low [24]. Barriers to care-seeking include embarrassment, the perception that symptoms are a normal part of aging, lack of awareness that effective treatments are available, and believing the symptoms do not warrant medical attention [25]. Therefore, the self-administered questionnaire includes direct questions that may initiate a conversation about symptoms that can be followed by appropriate treatment. The self-administered questionnaire was applied to 21 patients, and a series of issues (see Table 2) were identified.

## 4. Discussion

The self-administered questionnaire encompassed the physical, psychological, and social domains and is divided into categories.

### 4.1. Physical

1. Symptoms of breast cancer recurrence.
2. Physical sequelae of treatment.
3. Nutritional effects of treatment.
4. Prevention of recurrence.

### 4.2. Psychological

1. Psychological sequelae of diagnosis and treatment.
2. Symptoms of major depressive illness.

### 4.3. Social

1. Relationship problems consequent to diagnosis and treatment.
2. Sexual dysfunction.

Questions were designed to encourage proactive exploration of issues that may impact on the health and well-being of the patient. The Flesch reading ease test rates text on a 100-point scale [31]. The higher the score is, the easier it is to understand the document. For most standard files, the designers recommend a score between 60 and 70. Similarly, Flesh-Kincaid Grade test rates text on a US school grade level [32]. For example, a score of 8.0 means that an eighth grader, an early high school student, can understand the document [32]. It can also mean the number of years of education generally required to understand this text. For most documents, the aim is for a score of approximately 7.0 to 8.0. Therefore, the self-administered questionnaire was rated as easy to read and comprehend.

A key task in followup is to take the opportunity to assess for recurrent cancer. Alongside the plethora of physical symptoms and side effects, cancer can also result in a wide range of psychological and emotional problems which may contribute to greater patient distress [26]. The prevalence rates of distress in breast cancer patients are well documented and range from 35 to 49% in the USA and Europe [34]. There are few if any published self-administered questionnaires that can be used to help assess patients' needs following specialist treatment for chronic and complex conditions. Such self-completed self-administered questionnaires should be followed up with an interview and if necessary physical examination by a health practitioner with the necessary skill set.

The self-administered questionnaire detected symptoms or problems related to each domain and 121 individual items of unmet need despite the fact that the patients were already attending a hospital clinic and had access to their general practitioner. This emphasised the value of a proactive approach to the assessment of patients in the period following the treatment of breast cancer.

## 5. Conclusion

This self-administered screening tool may assist in identifying physical psychological and social dysfunction distress in women after breast cancer treatment. It contains screening questions focusing on psychological issues which may contribute to distress, including depression, anxiety, and general coping. The information collected from this screening tool, and elaborated on in the clinical interview with health professionals, should assist in identifying patients who are experiencing psychological distress and assist practitioners in recommending appropriate follow-up care plans. However the self-administered questionnaire needs to be further tested to confirm that it has predictive and concurrent validity. 

## Figures and Tables

**Table 1:** Justification for questions chosen per specialty.

Speciality	Questions	Rationale
Medicine	1–8, 23, 27, 28	Symptoms of recurrent breast cancer [13], depression [14], menopausal symptoms [15, 16], joint pain related to treatment [17]
Physiotherapy	9, 14–17, 24, 29	Shoulder pain [18], lymphoedema [18]
OT	18, 20	Adjustment and occupational issues [19]
Psychology	5, 19, 22, 25, 26, 27, 28	Depression, anxiety and general psychological well-being [20, 21], urinary incontinence [22–25], alcohol abuse as a risk factor for recurrence [21], undetected psychological distress [26]
Sexology	25, 29	Common sexual difficulties experienced following cancer treatment [27, 28]
Dietetics	5, 10, 11, 13, 20	Obesity as a risk of recurrence [29], motivation to maintain a healthy weight, physical activity as protection against recurrence [30]

**Table 2:** Problems identified on application of the self-administered questionnaire.

Domain	Type of problem	Number of patients/21
Physical	Upper body/arm dysfunction	14
	Weight management	9
	Urinary symptoms/incontinence	12
	Lower bowel symptoms	1
	Menopause symptoms	16
	Other physical symptoms	6
	Sleep disturbance	13

Psychological	Body image concerns	8
	Depression	6
	Other psychological distress	8
	Psychosexual problems	16

Social	Employment issues	5
	Financial concerns	7
